# Innate Immunity and Mobilization of Hematopoietic Stem Cells

**DOI:** 10.1007/s40778-017-0087-3

**Published:** 2017-07-10

**Authors:** Mateusz Adamiak, Mariusz Z. Ratajczak

**Affiliations:** 1 Stem Cell Institute at James Graham Brown Cancer Center, University of Louisville, 500 S. Floyd Street, Rm. 107, Louisville, KY 40202 USA; 2 Department of Regenerative Medicine, Warsaw Medical University, Warsaw, Poland

**Keywords:** Complement cascade, Hematopoietic stem cells, Stem cell mobilization, HO-1, iNOS

## Abstract

**Purpose of Review:**

Several mechanisms have been postulated to orchestrate mobilization of hematopoietic stem/progenitor cells (HSPCs), and still more work is needed to better understand this process and to gain better mechanistic insight.

**Recent Findings:**

Evidence accumulated that mobilization of HSPCs is a part of innate immunity response to tissue organ injury, stress, and infection. This evolutionary ancient process is orchestrated by granulocytes and monocytes that trigger activation of complement cascade and the coagulation cascade.

**Summary:**

We will present data from our laboratory that initiation of complement cascade activation and subsequently activation of the coagulation cascade during mobilization process are dependent on mannan-binding lectin (MBL). The mannan-binding pathway activates MBL-associated serine proteases (MASP-1 and MASP-2) that cleave the third complement component C3 and prothrombin. Cleavage of C3 leads to formation of classical C5 convertase and cleavage of prothrombin generates thrombin, which has “C5-like convertase” activity. Finally, both C5 convertase and thrombin cleave the fifth complement component C5, and activate distal part of the complement cascade that is crucial for egress of HSCPs from bone marrow niches into peripheral blood.

## Introduction

The alpha-chemokine stromal derived factor-1 (SDF-1)–C-X-C chemokine receptor type 4 (CXCR4) receptor and vascular adhesion molecule-1 (VCAM-1)–very late antigen-4 (VLA-4) integrin receptor axes play important roles in retention of hematopoietic stem/progenitor cells (HSPCs) in bone marrow (BM) niches [1, 2]. However, in steady-state conditions low numbers of HSPCs always circulate in peripheral blood (PB) and lymph, and undergo a circadian rhythm in their circulation, with the peak occurring early in the morning and the nadir at night [3, 4]. In addition to these circadian rhythms, the number of circulating HSPCs increases in PB in response to (i) systemic or local inflammation, (ii) strenuous exercise, (iii) hypoxia, and (iv) tissue/organ injuries [5, 6•, 7, 8•, 9].

Moreover, in clinical settings, administration of some agents may induce forced egress of HSPCs into PB and increase their number in PB up to 100-fold in a process known as “pharmacological mobilization.” Such drugs include cytokine granulocyte colony stimulating factor (G-CSF) and the small molecular CXCR4 antagonist AMD3100, also known as Plerixafor. The growth-related oncogene protein beta (Gro-β) [10, 11, 12••, 13, 14], some cytostatics (e.g., cyclophosphamide), and non-steroidal anti-inflammatory drugs could be employed as mobilizing agents as well [15–18]. Pharmacological mobilization is exploited as a means to obtain HSPCs for hematopoietic reconstitution in the clinical setting for mobilized peripheral blood (mPB) grafts and our previous research demonstrated that the complement cascade (ComC), as part of innate immunity and the evolutionary ancient response to tissue/organ injury and stress, is an important modulator of stem cell trafficking [12••, 13].

Understanding the mechanisms that govern mobilization of HSPCs is crucial for optimizing hematopoietic stem cell transplants using mPB grafts. Unfortunately, in autologous transplant settings, ~10% of normal patients and ~25% of patients after chemotherapy do not respond efficiently to currently recommended mobilization protocols and are deemed poor mobilizers [19•, 20, 21]. Therefore, it is important to develop more efficient mobilization protocols in order to harvest the required number of CD34^+^ HSPCs for transplantation.

In this review, we present data on a role of elements of innate immunity including (i) granulocytes, (ii) monocytes, (iii) naturally occurring antibodies (NAbs), and (iv) activation of ComC and coagulation cascade (CoaC) in mobilization of HSPCs.

## Chemoattractants for HSPCs and the Role of Accessory Cells Involved in Mobilization Process

Under steady-state conditions, HSPCs are actively retained in BM niches, and retention mechanisms counteract a continuous sphingosine-1-phosphate (S1P) chemoattractant gradient originating in blood plasma that can promote HSPCs egress into the blood, as recently demonstrated [22••, 23, 24]. The S1P concentration in PB is higher than in tissues as this active phosposphingolipid is transported by erythrocytes, albumins, and high-density lipoproteins (HDL) [25].

Overall, there have been very few chemoattractants identified for HSPCs besides S1P, the abovementioned alpha-chemokine SDF-1 [26•], ceramide-1-phosphate (C1P) [27], and some extracellular nucleotides such as ATP [28, 29]. The responsiveness of HSPCs to SDF-1 is modulated by several molecules related to innate immunity [12••, 30•, 31•] and inflammation [32, 33]. Migration of HSPCs is facilitated by certain cationic anti-microbial peptides [34]; innate immunity mediators, including ComC-derived C3 cleavage fragments C3a and _desArg_C3a; and cathelicidin LL-37, which enhance or prime the responsiveness of HSPCs to an SDF-1 gradient [32, 34, 35•, 36, 37, 38]. These findings demonstrate that the responsiveness of HSPCs to SDF-1 is modulated by several molecules related to innate immunity [12••, 30•, 31•] and inflammation [32, 33] As reported by other investigators, the responsiveness of HSPCs to an SDF-1 gradient may also be enhanced by the presence of prostaglandin E2 (PGE2) [11, 39] and independent from SDF-1, gradient HSPCs may also follow in the homing process of calcium-sensing mechanism [40]. HSPC migration has to be well balanced, and we have recently identified the potent intracellular negative regulators of cell migration including two anti-inflammatory enzymes: (i) heme oxygenase 1 (HO-1) [41, 42••] and (ii) inducible nitric oxide synthetase (iNOS) [43••]. These enzymes possess anti-inflammatory effects, and since mobilization of HSPCs is part of the inflammatory response, this explains the negative effect of HO-1 and iNOS on egress of HSPCs from BM into PB [41, 42••, 43••].

Several types of cells have also been described as being required for mobilization (e.g., granulocytes, monocytes, pericytes, CAR cells monomacs, and osteoclasts), and proteolytic enzymes are also required (e.g., metalloproteinases, elastase, cathepsins G and K) [19•]. Some controversies still persist about (i) the redundancy of proteolytic enzymes [44, 45] and (ii) the role of osteoclasts in mobilization [46, 47], and further studies are needed to resolve these issues. Recently, we have demonstrated in addition to proteolytic enzymes also some lipolytic enzymes such as phospholipase-beta 2 (PLC-β2) that are required for optimal mobilization of HSPCs [48].

## Elements of Innate Immunity and Their Relevance to Stem Cell Mobilization

**Fig. 1:**
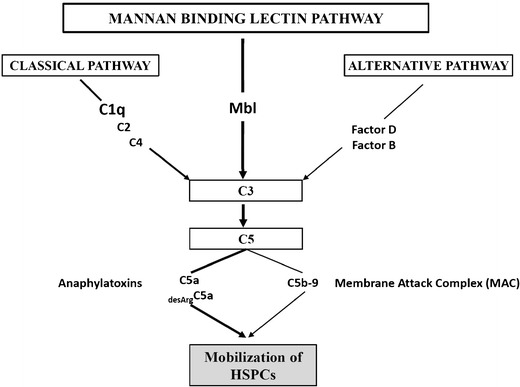
The mannan-binding lectin (MBL) pathway is crucial for triggering mobilization of HSPCs. ComC is activated by three pathways: (1) classical, (2) alternative, and (3) mannan-binding lectin pathways. Our previous studies excluded a role for the classical pathway of ComC activation in mobilization of *HSPCs*, as C1q KO mice do not show a defect in mobilization of these cells [29]. Recent research indicates a crucial role of mannan-binding lectin pathway. As shown at the scheme, the C5 cleavage fragments, anaphylatoxins *C5a* and _desArg_C5a, are crucial for egress of HSPCs from BM into PB by augmenting degranulation of granulocytes (release of proteolytic and lipolytic enzymes) and chemoattracting granulocytes and monocytes into PB. In addition, *C5b-C9* (*MAC*) may release S1P from erythrocytes and platelets and thus enhances the S1P level in PB, which directs egress of HSPCs. Of note, the alternative pathway is continuously active at a very low level under steady-state conditions. Contribution of this pathway to mobilization requires further studies

The innate (non-specific or inborn) immune system is an important part of host defense from infection by other organisms. The most important components of innate immunity are the ComC, NAbs, and Gr-1^+^ leucocytes. In addition, recent evidence indicates that ComC plays also an important pleiotropic role in embryonic development and tissue regeneration, which at least partly involve stem cell migration [49].The ComC is composed of several plasma proteins synthesized in the liver and is an important damage-sensing mechanism activated by the (i) classical, (ii) mannan-binding lectin (MBL), and (iii) alternative pathways (Fig. 1) [50–52]. NAbs are produced without any previous infection, vaccination, or foreign-antigen exposure. In addition to B cell derived NAbs, monocytes and neutrophils are also involved in mobilization of HSPCs [53, 54].

An important role in innate immunity is also played by pattern-recognition receptors (PRRs) [55, 56, 57, 58••]. These proteins are expressed on the surface of cells of the innate immune system or circulate free in PB to identify and bind to two classes of molecules: (i) pathogen-associated molecular patterns (PAMPs), which are expressed by microbial pathogens and what is relevant to this review, and (ii) damage-associated molecular patterns (DAMPs), which are associated with cell components and are released during cell activation, cell damage, or cell death [50, 56, 57, 58••].

PRRs are classified according to their ligand specificity, function, localization, and/or evolutionary relationships and may be divided into cell membrane PRRs (toll-like receptors, TLRs) and intracytoplasmic PRRs (NOD-like receptors and RIG-I-like receptors) [50, 55, 56, 57, 58••, 59]. Our most recent data demonstrated that TLRs play a negative role in mobilization, as they upregulate anti-inflammatory HO-1 activity in HSPCs that as mentioned above inhibits cell migration and mobilization process [60].

We became interested in other PRRs that are secreted and circulate in blood (e.g., collectins and ficolins). A collectin, mannose-binding lectin (MBL), is a major PRR of the innate immune system that binds to a wide range of pathogens [51, 52, 61]. MBL also binds phospholipids and several DAMPs released from activated cells, such as high-mobility group box 1 (HMGB1), extracellular ATP, DNA, and hyaluronan fragments [55, 56, 59, 62]. Once bound to ligands, MBL recruits MBL-associated serine proteases (MASP-1 and MASP-2) [51, 62, 63] and initiates activation of the ComC. After cleavage of C3, the next step is generation of classical C5 convertase that cleaves C5 into C5a and iC5b. In parallel, MASPs also activate prothrombin that gives rise to thrombin which has C5 “convertase-like” activity. C5 cleavage fragments [64•] are crucial for egress of HSPCs from BM, and this process is negatively regulated by HO-1 and iNOS, as we recently reported [41, 42••, 43••]. The fact that MBL–MASP interaction simultaneously activates coagulation (CoA) cascade explains the observed crosstalk between both cascades in HSPCs mobilization process [65••, 66••, 67].

## A Novel View of ComC-Related Pro-mobilizing Mechanisms—Turning Away from the Classical to Implicate the Mannan-Binding Pathway for ComC Cascade Activation

We are aware that, since mobilization of HSPCs is an evolutionarily ancient and important biological process, there may be redundant mechanisms that modulate this process. Nevertheless, elements of innate immunity, such as ComC activation, granulocytes, monocytes, and NAbs, play major roles.

When we initially discovered a requirement for ComC activation in HSPC mobilization, we assumed that the classical activation pathway of ComC would play a pivotal role [12••]. However, to our surprise, mice with mutations in components that initiate the classical pathway (e.g., C1q^−/−^ mice) (Fig. 1) do not show impairment in mobilization of HSPCs [68]. Thus, the main question emerged, which ComC activation pathway triggers mobilization of HSPCs after administration of G-CSF or AMD3100? We recently demonstrated that it is likely to be the aforementioned MBL pathway that comes into play Fig. 1. Specifically, mice deficient in MBL and MASP-1 are poor mobilizers [66••]. Moreover, by contrast, mobilization is enhanced in mice HO-1- and iNOS-deficient animals [42••, 43••] indicating that both these enzymes are negative counterbalancing regulators of this process.

**Fig. 2:**
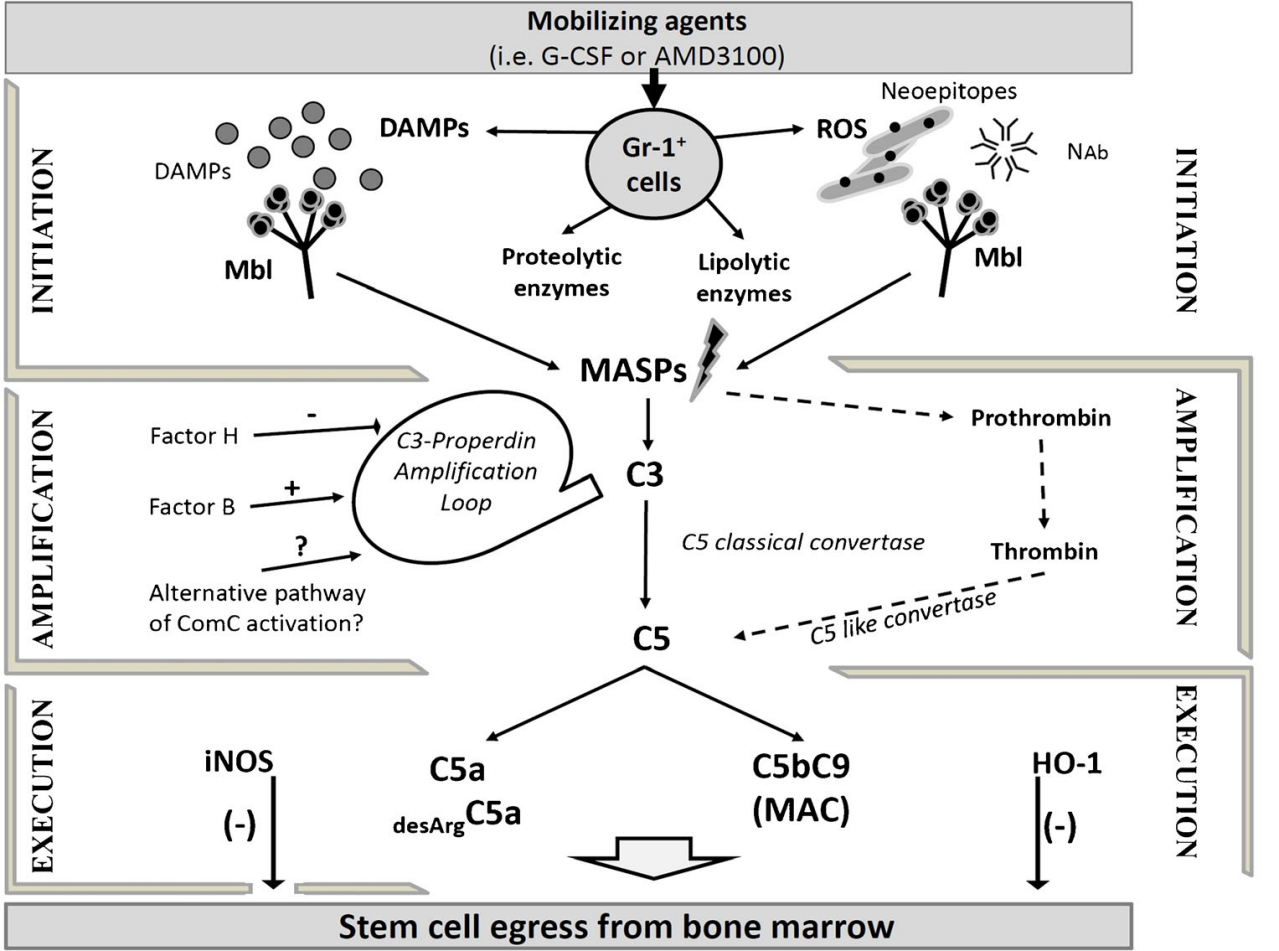
Proposed three-step model for triggering mobilization of HSPCs. All the phases of mobilization process are depicted. Step 1 (*initiation* phase). Activation of Gr-1^+^ neutrophils and monocytes by mobilizing agent (e.g., *G-CSF* or *AMD3100*) induces secretion of ROS and DAMPs by these cells. In the BM microenvironment, ROS expose neoepitopes. Neoepitope–IgM complexes and DAMPs are recognized by MBL, which activates the ComC and CoaC in a MASP-dependent manner. Step 2 (*amplification*). Convertases (classical C5 and C5-like) generated in this step cleave C5 to release cleavage fragments crucial in egress of HSPCs from BM. This step is also modulated by a C3 auto-amplification loop, with a possible contribution from the alternative ComC activation pathway. Step 3 (*execution*). In this step, C5 cleavage fragments promote release of HSPCs from BM, and this process is negatively regulated by HO-1 and iNOS—shown as negative sign

To provide a step-by-step mechanistic picture (Fig. 2), we propose that activation of Gr-1^+^ granulocytes and monocytes by mobilizing agents leads to the release of proteolytic enzymes [69••] and lipolytic enzymes [48] that disrupt the SDF-1–CXCR4 and VCAM-1–VLA-4 retention axes that keep HSPCs in their BM niches and to the secretion of reactive oxygen species (ROS) that expose auto-antigens known as “neoepitopes” in the BM microenvironment, which bind NAbs, mainly of the IgM class [37, 62]. It is known that NAbs that are produced as mentioned above without any previous infection, vaccination, other foreign antigen exposure, or passive immunization may preferentially recognize oxidation-specific epitopes exposed by ROS during inflammation [62, 66••].

The binding of NAbs to neoepitopes creates a damage-associated molecular pattern (DAMP) complex that is recognized by MBL, being a major soluble pattern-recognition receptor (PRR) of the innate immune system that triggers activation of the ComC (Fig. 2). In addition, Gr-1^+^ cells also release other soluble DAMPs that are recognized by MBL including high-mobility group box 1 (HMGB1), extracellular ATP, DNA, and hyaluronan fragments [55, 56, 59, 62]. Our preliminary data show that all these molecules are potentiating mobilization in MBL-MASP-dependent manner.

In this context, it is important to more thoroughly explore the role of the ComC in modulating the function of Gr-1^+^ cells (granulocytes and monocytes) in BM, as they are the first cells that egress from BM into PB and pave the way for HSPCs to cross the BM–PB endothelial barrier [12••, 54, 70, 71••]. This occurs in response to increase in C5a level in PB due to ComC activation. C5a is a most potent activator and chemoattractant for Gr-1^+^ cells [12••]. In addition, it has been postulated that monocytes may secrete factors that inhibit SDF-1 expression in BM niches [70, 72].

Based on these considerations, we propose the following involvement of innate immunity in the mobilization of HSPCs and distinguish three main phases of this process: (i) initiation, (ii) amplification, and (iii) execution phase (Fig. 2). These three phases will be shortly discussed below.

### Initiation Phase

Mobilizing agents G-CSF or AMD3100 (which besides its blocking properties is also a partial agonist of CXCR4) [73] activate Gr-1^+^ neutrophils and monocytes and enhance secretion of reactive oxygen species (ROS) by these cells. In the BM microenvironment, ROS expose neoepitopes on BM cells. Moreover, during mobilization there are released from BM cells several danger-associated molecular pattern (DAMP) molecules such as HMGB1, extracellular ATP, DNA, and hyaluronan fragments. Both neoepitope–IgM complexes and released DAMPs are recognized by MBL, which activates the ComC and CoaC in a MASP-dependent manner [66••].

### Amplification Phase

Convertases (classical C5 and C5-like) generated in this step cleave C5 to release cleavage fragments C5a and iC5b that are crucial in egress of HSPCs from BM. This step is also modulated by a C3 auto-amplification loop, with a possible contribution from the alternative ComC activation pathway. To support involvement of this pathway, MBL^−/−^ and MASP-1^−/−^ mice still mobilize HSPCs, however in much less effective manner. Overall, the alternative pathway is activated by foreign or the organism’s own damaged cells and is facilitated by the continuous spontaneous hydrolysis of C3. The alternative pathway of ComC activation also has an important function by providing an amplification loop enhancing C3 activation, independent of which ComC pathway was initially activated. This effect is mainly due to properdin, the only positive regulator in the complement system, which stabilizes C3 convertase. To confirm involvement of alternative ComC activation pathway, it would be important to perform mobilization studies in B factor- and properdin- and factor H-deficient mice, as these factors regulate the spontaneous C3 amplification loop. Impaired mobilization in these animals would support involvement of alternative pathway of ComC activation.

### Execution Step

In this step, C5 cleavage fragments promote release of HSPCs from BM, and this process is negatively regulated by HO-1 and iNOS. To support this later notion, activation of ComC-mediated mobilization must be strictly controlled, and our most recent research revealed that both HO-1 and iNOS are negative regulators of cell trafficking that attenuate mobilization process [41, 42••, 43••]. HO-1 is an inducible stress–response enzyme that not only catalyzes the degradation of heme (e.g., released from erythrocytes) but also has an important function in various states associated with cellular stress [74–76]. Inherited HO-1 deficiency in humans and in mice results in vulnerability to stressful injury and inflammation, which can be explained by hyperactivity of the ComC [77].

We demonstrated for the first time that HO-1 plays an important and heretofore unrecognized role in retention of HSPCs in BM niches by (i) negatively modulating activation of the mobilization-promoting ComC and (ii) attenuating the chemotaxis of HSPCs in response to SDF-1 and S1P gradients [41, 42••]. Furthermore, our results showing a positive mobilizing effect by a non-toxic, small-molecule inhibitor of HO-1 (SnPP) suggest that blockade of HO-1 would be a promising strategy to facilitate mobilization of HSPCs in so-called poor mobilizers [42••]. Thus, this observation is highly relevant for developing more efficient mobilization strategies for HSPCs. Moreover, iNOS is also an inducible stress-response enzyme and produces nitric oxide (NO), which is a gaseous free radical molecule involved in several biological processes related to inflammation, tissue damage, and infections, and iNOS activity is enhanced during ComC activation as well [78]. Similarly, we found that inhibition of iNOS in HSPCs by the small-molecule inhibitor NIL also enhances mobilization of HSPCs. We propose again that these simple and inexpensive strategies to inhibit iNOS could be employed in poor mobilizers similarly as HO-1 inhibition to enhance process of HSPC mobilization [43••].

## Does an Inherited Defective Activation of the Mbl in Patients Explain Poor Mobilization Status?

Since MBL ComC activation pathway plays a crucial role in triggering mobilization of HSPCs, one can ask if MBL deficiency may explain the poor mobilization status of some patients. Human MBL (MBL2) deficiency, the most common form of complement deficiency, is seen in 5–10% of the population, and correlates with the percentage of poor mobilizers. In humans, MBL is produced in the liver, and structural mutations in exon 1 of the human *MBL2* gene at codon 52 (Arg → Cys, allele D), codon 54 (Gly → Asp, allele B), and codon 57 (Gly → Glu, allele C) independently reduce the level of functional serum MBL by disrupting the protein structure. Furthermore, several nucleotide substitutions in the promoter region of the human *MBL2* gene at positions −550 (H/L polymorphism); −221 (X/Y polymorphism); and −427, −349, −336, del (−324 to −329), −70, and +4 (P/Q polymorphisms) affect MBL2 serum levels [79–81]. It remains to be shown whether MBL2 would be a good biomarker to predict poor mobilization; this concept is worth pursuing as could help to predict outcome of mobilization in HSPCs donors.

## Conclusions

Optimization of stem cell mobilization is an important goal to obtain optimal graft that could significantly improve clinical outcomes following transplantation. Our data indicate (i) the crucial role of MBL–MASP in triggering ComC-directed trafficking of HSPCs, (ii) presence of functional crosstalk between the ComC and the CoA, and (iii) identify HO-1 and iNOS as negative regulators of ComC-mediated mobilization processes. Our observations are also highly relevant for other processes in which an increase in stem cell trafficking is observed in response to stress related to infection, tissue/organ injury, or strenuous exercise. Thus, these observations by modulating innate immunity responses will enable development of innovative treatment approaches, not only in hematopoietic settings but also when stem cells are employed to treat tissue/organ injuries, as seen in regenerative medicine.

## References

[1] Levesque JP, Takamatsu Y, Nilsson SK, Haylock DN, Simmons PJ (2001). Vascular cell adhesion molecule-1 (CD106) is cleaved by neutrophil proteases in the bone marrow following hematopoietic progenitor cell mobilization by granulocyte colony-stimulating factor. Blood.

[2] Peled A, Grabovsky V, Habler L, Sandbank J, Arenzana-Seisdedos F, Petit I (1999). The chemokine SDF-1 stimulates integrin-mediated arrest of CD34(+) cells on vascular endothelium under shear flow. J Clin Invest.

[3] Giudice A, Caraglia M, Marra M, Montella M, Maurea N, Abbruzzese A (2010). Circadian rhythms, adrenergic hormones and trafficking of hematopoietic stem cells. Expert Opin Ther Targets.

[4] Mendez-Ferrer S, Chow A, Merad M, Frenette PS (2009). Circadian rhythms influence hematopoietic stem cells. Curr Opin Hematol.

[5] Luster AD, Alon R, von Andrian UH (2005). Immune cell migration in inflammation: present and future therapeutic targets. Nat Immunol.

[6] Simón MF, Andrew C, Miriam M, Paul SF (2009). Circadian rhythms influence hematopoietic stem cells. Curr Opin Hematol.

[7] Möbius-Winkler S, Hilberg T, Menzel K, Golla E, Burman A, Schuler G (2009). Time-dependent mobilization of circulating progenitor cells during strenuous exercise in healthy individuals. J Appl Physiol.

[8] Massberg S, Schaerli P, Knezevic-Maramica I, Köllnberger M, Tubo N, Moseman EA (2007). Immunosurveillance by hematopoietic progenitor cells trafficking through blood, lymph, and peripheral tissues. Cell.

[9] Wojakowski W, Tendera M, Kucia M, Zuba-Surma E, Paczkowska E, Ciosek J (2009). Mobilization of bone marrow-derived Oct-4+ SSEA-4+ very small embryonic-like stem cells in patients with acute myocardial infarction. J Am Coll Cardiol.

[10] Lee HM, Wysoczynski M, Liu R, Shin DM, Kucia M, Botto M (2010). Mobilization studies in complement-deficient mice reveal that optimal AMD3100 mobilization of hematopoietic stem cells depends on complement cascade activation by AMD3100-stimulated granulocytes. Leukemia.

[11] Hoggatt J, Pelus LM (2010). Eicosanoid regulation of hematopoiesis and hematopoietic stem and progenitor trafficking. Leukemia.

[12] Lee HM, Wu W, Wysoczynski M, Liu R, Zuba-Surma EK, Kucia M (2009). Impaired mobilization of hematopoietic stem/progenitor cells in C5-deficient mice supports the pivotal involvement of innate immunity in this process and reveals novel promobilization effects of granulocytes. Leukemia.

[13] Wysoczynski M, Reca R, Ratajczak J, Kucia M, Shirvaikar N, Honczarenko M (2005). Incorporation of CXCR4 into membrane lipid rafts primes homing-related responses of hematopoietic stem/progenitor cells to an SDF-1 gradient. Blood.

[14] Pelus LM, Fukuda S (2006). Peripheral blood stem cell mobilization: the CXCR2 ligand GRObeta rapidly mobilizes hematopoietic stem cells with enhanced engraftment properties. Exp Hematol.

[15] Wright DE, Cheshier SH, Wagers AJ, Randall TD, Christensen JL, Weissman IL (2001). Cyclophosphamide/granulocyte colony-stimulating factor causes selective mobilization of bone marrow hematopoietic stem cells into the blood after M phase of the cell cycle. Blood.

[16] Winkler IG, Pettit AR, Raggatt LJ, Jacobsen RN, Forristal CE, Barbier V (2012). Hematopoietic stem cell mobilizing agents G-CSF, cyclophosphamide or AMD3100 have distinct mechanisms of action on bone marrow HSC niches and bone formation. Leukemia.

[17] Hoggatt J, Mohammad KS, Singh P, Hoggatt AF, Chitteti BR, Speth JM (2013). Differential stem- and progenitor-cell trafficking by prostaglandin E2. Nature.

[18] Marshall E, Woolford LB, Lord BI (1997). Continuous infusion of macrophage inflammatory protein MIP-1alpha enhances leucocyte recovery and haemopoietic progenitor cell mobilization after cyclophosphamide. Br J Cancer.

[19] Hoggatt J, Pelus LM (2011). Many mechanisms mediating mobilization: an alliterative review. Curr Opin Hematol.

[20] Eide MB, Lauritzsen GF, Kvalheim G, Kolstad A, Fagerli UM, Maisenhölder M (2011). High dose chemotherapy with autologous stem cell support for patients with histologically transformed B-cell non-Hodgkin lymphomas. A Norwegian multi centre phase II study. Br J Haematol.

[21] Girbl T, Lunzer V, Greil R, Namberger K, Hartmann TN (2014). The CXCR4 and adhesion molecule expression of CD34+ hematopoietic cells mobilized by “on-demand” addition of plerixafor to granulocyte–colony-stimulating factor. Transfusion.

[22] Ratajczak MZ, Lee H, Wysoczynski M, Wan W, Marlicz W, Laughlin MJ (2010). Novel insight into stem cell mobilization-plasma sphingosine-1-phosphate is a major chemoattractant that directs the egress of hematopoietic stem progenitor cells from the bone marrow and its level in peripheral blood increases during mobilization due to activation of complement cascade/membrane attack complex. Leukemia.

[23] Golan K, Vagima Y, Ludin A, Itkin T, Cohen-Gur S, Kalinkovich A (2012). S1P promotes murine progenitor cell egress and mobilization via S1P1-mediated ROS signaling and SDF-1 release. Blood.

[24] Juarez JG, Harun N, Thien M, Welschinger R, Baraz R, Pena AD (2012). Sphingosine-1-phosphate facilitates trafficking of hematopoietic stem cells and their mobilization by CXCR4 antagonists in mice. Blood.

[25] Potì F, Simoni M, Nofer JR (2014). Atheroprotective role of high-density lipoprotein (HDL)-associated sphingosine-1-phosphate (S1P). Cardiovasc Res.

[26] Lapidot T, Dar A, Kollet O (2005). How do stem cells find their way home?. Blood.

[27] Ratajczak MZ, Suszynska M, Borkowska S, Ratajczak J, Schneider G (2014). The role of sphingosine-1-phosphate (S1P) and ceramide-1-phosphate (C1P) in the trafficking of normal and malignant cells. Expert Opin Ther Targets.

[28] Lemoli RM, Ferrari D, Fogli M, Rossi L, Pizzirani C, Forchap S (2004). Extracellular nucleotides are potent stimulators of human hematopoietic stem cells in vitro and in vivo. Blood.

[29] Rossi L, Manfredini R, Bertolini F, Ferrari D, Fogli M, Zini R (2007). The extracellular nucleotide UTP is a potent inducer of hematopoietic stem cell migration. Blood.

[30] Wu W, Kim CH, Liu R, Kucia M, Marlicz W, Greco N (2012). The bone marrow-expressed antimicrobial cationic peptide LL-37 enhances the responsiveness of hematopoietic stem progenitor cells to an SDF-1 gradient and accelerates their engraftment after transplantation. Leukemia.

[31] Hoggatt J, Singh P, Sampath J, Pelus LM (2009). Prostaglandin E2 enhances hematopoietic stem cell homing, survival, and proliferation. Blood.

[32] Adams GB, Chabner KT, Alley IR, Olson DP, Szczepiorkowski ZM, Poznansky MC (2006). Stem cell engraftment at the endosteal niche is specified by the calcium-sensing receptor. Nature.

[33] Ratajczak MZ, Reca R, Wysoczynski M, Kucia M, Baran JT, Allendorf DJ (2004). Transplantation studies in C3-deficient animals reveal a novel role of the third complement component (C3) in engraftment of bone marrow cells. Leukemia.

[34] Lee H, Ratajczak M (2009). Innate immunity: a key player in the mobilization of hematopoietic stem/progenitor cells. Arch Immunol Ther Exp.

[35] Reca R, Mastellos D, Majka M, Marquez L, Ratajczak J, Franchini S (2003). Functional receptor for C3a anaphylatoxin is expressed by normal hematopoietic stem/progenitor cells, and C3a enhances their homing-related responses to SDF-1. Blood.

[36] Ratajczak MZ, Kim CH, Wojakowski W, Janowska-Wieczorek A, Kucia M, Ratajczak J (2010). Innate immunity as orchestrator of stem cell mobilization. Leukemia.

[37] Schuettpelz LG, Borgerding JN, Christopher MJ, Gopalan PK, Romine MP, Herman AC (2014). G-CSF regulates hematopoietic stem cell activity, in part, through activation of Toll-like receptor signaling. Leukemia.

[38] Kim CH, Wu W, Wysoczynski M, Abdel-Latif A, Sunkara M, Morris A (2012). Conditioning for hematopoietic transplantation activates the complement cascade and induces a proteolytic environment in bone marrow: a novel role for bioactive lipids and soluble C5b-C9 as homing factors. Leukemia.

[39] Wysoczynski M, Reca R, Lee H, Wu W, Ratajczak J, Ratajczak MZ (2009). Defective engraftment of C3aR−/− hematopoietic stem progenitor cells shows a novel role of the C3a-C3aR axis in bone marrow homing. Leukemia.

[40] Ratajczak MZ, Reca R, Wysoczynski M, Yan J, Ratajczak J (2006). Modulation of the SDF-1-CXCR4 axis by the third complement component (C3)-implications for trafficking of CXCR4+ stem cells. Exp Hematol.

[41] Adamiak M, Moore JB, Zhao J, Abdelbaset-Ismail A, Grubczak K, Rzeszotek S (2016). Downregulation of heme oxygenase 1 (HO-1) activity in hematopoietic cells enhances their engraftment after transplantation. Cell Transplant.

[42] Wysoczynski M, Ratajczak J, Pedziwiatr D, Rokosh G, Bolli R, Ratajczak MZ (2015). Identification of heme oxygenase 1 (HO-1) as a novel negative regulator of mobilization of hematopoietic stem/progenitor cells. Stem Cell Rev.

[43] •• Adamiak M, Abdelbaset-Ismail A, Moore JB 4th, Zhao J, Abdel-Latif A, Wysoczynski M, et al. Inducible nitric oxide synthase (iNOS) is a novel negative regulator of hematopoietic stem/progenitor cell trafficking. Stem Cell Rev. 2016; doi:10.1007/s12015-016-9693-1. **This paper provides an evidence that inducible nitric oxide synthase expressed in hematopoietic cells inhibits mobilization process.** 10.1007/s12015-016-9693-1 PMC5346113 27752990

[44] Kollet O, Dar A, Shivtiel S, Kalinkovich A, Lapid K, Sztainberg Y (2006). Osteoclasts degrade endosteal components and promote mobilization of hematopoietic progenitor cells. Nat Med.

[45] Adamiak M, Poniewierska-Baran A, Borkowska S, Schneider G, Abdelbaset-Ismail A, Suszynska M (2016). Evidence that a lipolytic enzyme—hematopoietic-specific phospholipase C-beta2—promotes mobilization of hematopoietic stem cells by decreasing their lipid raft-mediated bone marrow retention and increasing the promobilizing effects of granulocytes. Leukemia.

[46] Cho SY, Xu M, Roboz J, Lu M, Mascarenhas J, Hoffman R (2010). The effect of CXCL12 processing on CD34+ cell migration in myeloproliferative neoplasms. Cancer Res.

[47] Levesque JP, Hendy J, Takamatsu Y, Simmons PJ, Bendall LJ (2003). Disruption of the CXCR4/CXCL12 chemotactic interaction during hematopoietic stem cell mobilization induced by GCSF or cyclophosphamide. J Clin Invest.

[48] Chang MK, Raggatt LJ, Alexander KA, Kuliwaba JS, Fazzalari NL, Schroder K (2008). Osteal tissue macrophages are intercalated throughout human and mouse bone lining tissues and regulate osteoblast function in vitro and in vivo. J Immunol.

[49] Ratajczak MZ, Zuba-Surma E, Kucia M, Reca R, Wojakowski W, Ratajczak J (2006). The pleiotropic effects of the SDF-1–CXCR4 axis in organogenesis, regeneration and tumorigenesis. Leukemia.

[50] Degn SE, Thiel S (2013). Humoral pattern recognition and the complement system. Scand J Immunol.

[51] Heja D, Kocsis A, Dobo J, Szilagyi K, Szasz R, Zavodszky P (2012). Revised mechanism of complement lectin-pathway activation revealing the role of serine protease MASP-1 as the exclusive activator of MASP-2. Proc Natl Acad Sci U S A.

[52] Ip WK, Takahashi K, Ezekowitz RA, Stuart LM (2009). Mannose-binding lectin and innate immunity. Immunol Rev.

[53] Travnickova J, Tran Chau V, Julien E, Mateos-Langerak J, Gonzalez C, Lelievre E (2015). Primitive macrophages control HSPC mobilization and definitive haematopoiesis. Nat Commun.

[54] Singh P, Hu P, Hoggatt J, Moh A, Pelus LM (2012). Expansion of bone marrow neutrophils following G-CSF administration in mice results in osteolineage cell apoptosis and mobilization of hematopoietic stem and progenitor cells. Leukemia.

[55] Burberry A, Zeng MY, Ding L, Wicks I, Inohara N, Morrison SJ (2014). Infection mobilizes hematopoietic stem cells through cooperative NOD-like receptor and Toll-like receptor signaling. Cell Host Microbe.

[56] Kang JW, Kim SJ, Cho HI, Lee SM (2015). DAMPs activating innate immune responses in sepsis. Ageing Res Rev.

[57] Pandey S, Singh S, Anang V, Bhatt AN, Natarajan K, Dwarakanath BS (2015). Pattern recognition receptors in cancer progression and metastasis. Cancer Growth Metastasis.

[58] Schuettpelz LG, Link DC (2013). Regulation of hematopoietic stem cell activity by inflammation. Front Immunol.

[59] Mogensen TH (2009). Pathogen recognition and inflammatory signaling in innate immune defenses. Clin Microbiol Rev.

[60] Yanez A, Goodridge HS, Gozalbo D, Gil ML (2013). TLRs control hematopoiesis during infection. Eur J Immunol.

[61] Adamiak M, Abdelbaset-Ismail A, Kucia M, Ratajczak J, Ratajczak MZ (2016). Toll-like receptor (TLR) signaling-deficient mice are easy mobilizers—evidence that TLR signaling prevents mobilization of hematopoietic stem/progenitor cells in HO-1-dependent manner. Leukemia.

[62] Joseph K, Kulik L, Coughlin B, Kunchithapautham K, Bandyopadhyay M, Thiel S (2013). Oxidative stress sensitizes retinal pigmented epithelial (RPE) cells to complement-mediated injury in a natural antibody-, lectin pathway-, and phospholipid epitope-dependent manner. J Biol Chem.

[63] Gadjeva M, Thiel S, Jensenius JC. Assays for the mannan-binding lectin pathway. Curr Protoc Immunol. 2004;Chapter 13:Unit 13. doi:10.1002/0471142735.im1306s58. 10.1002/0471142735.im1306s58 18432925

[64] Jalili A, Shirvaikar N, Marquez-Curtis L, Qiu Y, Korol C, Lee H (2010). Fifth complement cascade protein (C5) cleavage fragments disrupt the SDF-1/CXCR4 axis: further evidence that innate immunity orchestrates the mobilization of hematopoietic stem/progenitor cells. Exp Hematol.

[65] Borkowska S, Suszynska M, Mierzejewska K, Ismail A, Budkowska M, Salata D (2014). Novel evidence that crosstalk between the complement, coagulation and fibrinolysis proteolytic cascades is involved in mobilization of hematopoietic stem/progenitor cells (HSPCs). Leukemia.

[66] Adamiak M, Abdelbaset-Ismail A, Suszynska M, Abdel-Latif A, Ratajczak J, Ratajczak MZ (2017). Novel evidence that the mannan-binding lectin pathway of complement activation plays a pivotal role in triggering mobilization of hematopoietic stem/progenitor cells by activation of both the complement and coagulation cascades. Leukemia.

[67] Gur-Cohen S, Itkin T, Chakrabarty S, Graf C, Kollet O, Ludin A (2015). PAR1 signaling regulates the retention and recruitment of EPCR-expressing bone marrow hematopoietic stem cells. Nat Med.

[68] Jalili A, Marquez-Curtis L, Shirvaikar N, Wysoczynski M, Ratajczak M, Janowska-Wieczorek A. Complement C1q enhances homing-related responses of hematopoietic stem/progenitor cells. Transfusion. 2010;50:2002–10. doi:10.1111/j.1537-2995.2010.02664.x. 10.1111/j.1537-2995.2010.02664.x PMC2974804 20456695

[69] Levesque JP, Hendy J, Takamatsu Y, Williams B, Winkler IG, Simmons PJ (2002). Mobilization by either cyclophosphamide or granulocyte colony-stimulating factor transforms the bone marrow into a highly proteolytic environment. Exp Hematol.

[70] Christopher MJ, Rao M, Liu F, Woloszynek JR, Link DC (2011). Expression of the G-CSF receptor in monocytic cells is sufficient to mediate hematopoietic progenitor mobilization by G-CSF in mice. J Exp Med.

[71] Pruijt JF, Verzaal P, van Os R, de Kruijf EJ, van Schie ML, Mantovani A (2002). Neutrophils are indispensable for hematopoietic stem cell mobilization induced by interleukin-8 in mice. Proc Natl Acad Sci U S A.

[72] Winkler IG, Sims NA, Pettit AR, Barbier V, Nowlan B, Helwani F (2010). Bone marrow macrophages maintain hematopoietic stem cell (HSC) niches and their depletion mobilizes HSCs. Blood.

[73] Zhang WB, Navenot JM, Haribabu B, Tamamura H, Hiramatu K, Omagari A (2002). A point mutation that confers constitutive activity to CXCR4 reveals that T140 is an inverse agonist and that AMD3100 and ALX40-4C are weak partial agonists. J Biol Chem.

[74] Cao YA, Wagers AJ, Karsunky H, Zhao H, Reeves R, Wong RJ (2008). Heme oxygenase-1 deficiency leads to disrupted response to acute stress in stem cells and progenitors. Blood.

[75] Freitas A, Alves-Filho JC, Secco DD, Neto AF, Ferreira SH, Barja-Fidalgo C (2006). Heme oxygenase/carbon monoxide-biliverdin pathway down regulates neutrophil rolling, adhesion and migration in acute inflammation. Br J Pharmacol.

[76] Paine A, Eiz-Vesper B, Blasczyk R, Immenschuh S (2010). Signaling to heme oxygenase-1 and its anti-inflammatory therapeutic potential. Biochem Pharmacol.

[77] Kawashima A, Oda Y, Yachie A, Koizumi S, Nakanishi I (2002). Heme oxygenase-1 deficiency: the first autopsy case. Hum Pathol.

[78] Bogdan C (2001). Nitric oxide and the immune response. Nat Immunol.

[79] Holdaway J, Deacock S, Williams P, Karim Y (2016). Mannose-binding lectin deficiency and predisposition to recurrent infection in adults. J Clin Pathol.

[80] Keizer MP, Wouters D, Schlapbach LJ, Kuijpers TW (2014). Restoration of MBL-deficiency: redefining the safety, efficacy and viability of MBL-substitution therapy. Mol Immunol.

[81] Reis ES, Lange T, Kohl G, Herrmann A, Tschulakow AV, Naujoks J (2011). Sleep and circadian rhythm regulate circulating complement factors and immunoregulatory properties of C5a. Brain Behav Immun.

